# Conceptualisation of health inequalities by local healthcare systems: A document analysis

**DOI:** 10.1111/hsc.13791

**Published:** 2022-03-30

**Authors:** Jasmine N. Olivera, John Ford, Sarah Sowden, Clare Bambra

**Affiliations:** ^1^ Department of Public Health and Primary Care Jesus College University of Cambridge Cambridge UK; ^2^ 2152 Department of Public Health and Primary Care University of Cambridge Cambridge UK; ^3^ Population Health Sciences Institute Newcastle University Newcastle upon Tyne UK

**Keywords:** health inequalities, health policy, healthcare, quality, access and evaluation, inequalities in health and healthcare, patient care management, public health systems’ research

## Abstract

In 2019, local healthcare systems in England were asked to develop formal plans to reduce health inequalities. Here, we explore plans to understand how local healthcare systems conceptualise health inequalities and why. A broad Internet search and targeted search of NHS websites were conducted to identify all publicly accessible healthcare planning documents (National Health Service (NHS) Long‐Term Plan (LTP) response documents) produced by local health partnerships in England. A thematic document analysis of the accessible plans was undertaken in NVivo by coding text relating to health inequalities. Of the 44 documents developed, 13 were publicly accessible. These 13 local plans were submitted to NHS England for review between September 2019 and January 2020 and averaged 167 pages (range: 41–273 pages). Only one document contained a chapter dedicated to health inequalities. After analysis, five themes were identified: (1) v*ariation* and (2) *vagueness* explained how health inequalities were conceptualised and (3) u*se of value judgements, (4) lack of prior conceptualisation and approach* and (5) a *lack of commitment to action* in the documents to reduce health inequalities explained what led to the overall vagueness and variation. Local healthcare systems were found to conceptualise health inequalities in a vague and varying manner, and their conceptualisations did not reflect established health inequalities frameworks. A clear conceptual national framework for addressing health inequalities is needed to support local healthcare systems, so they can address health inequalities meaningfully and sustainably.

**What is known about this topic?**

- Inequalities in health outcomes and care are widening in many instances, particularly as a result of the COVID‐19 pandemic.
- Healthcare systems are being increasingly tasked with addressing health inequalities.
- There is currently little research exploring how local healthcare systems conceptualise and approach health inequalities.

**What this paper adds?**

- Based on planning documents, local healthcare systems in England were found to conceptualise and approach health inequalities in a manner that was vague and inconsistent.
- There appeared to be a use of value judgements, lack of prior conceptualisation and a lack of commitment to concrete action to reduce inequalities.
- A clear conceptual national framework for addressing health inequalities is needed to support local healthcare systems.

## 1 INTRODUCTION

Despite health inequalities being a priority for many countries, the gap in access and quality of healthcare and health outcomes between the most and least disadvantaged groups is widening in many cases (Ford et al., 2019; Graham & Kelly, 2004; Killoran & Kelly, 2004). The COVID‐19 pandemic has further exacerbated health inequalities (Bambra et al., 2020; Public Health England, 2020). Healthcare organisations specifically are increasingly being asked to take action to address inequalities (National Health Service (NHS), 2019). For example, in 2012, the Health and Social Care Act was passed in England, introducing the first legal duties for healthcare organisations to address inequalities in access and outcomes (NHS England, 2020). In 2018, the UK government pledged £20 billion to the NHS in England, contingent on developing a spending plan. The result was the Long‐Term Plan (LTP), published in 2019, which ‘[set] out the pathway for a new service model fit for the 21st century’ (NHS, 2019). Health inequalities were a priority in the LTP, which asked local systems to develop their own plans that contain ‘specific measurable goals and mechanisms by which they will contribute to narrowing health inequalities over the next 5 and 10 years’ (NHS, 2019). These local healthcare plans were submitted to NHS England in fall 2019 for feedback, with the goal of publishing final versions by spring 2020.

The LTP itself has been criticised for lacking clarity in defining risk factors of health inequality and populations affected by them, as well as for its goals to reduce health inequalities lacking in direction (Ford et al., 2019). It is also lacking in specific metrics and indicators that local healthcare partnerships can use to track health inequalities. According to Regmi and Mudyarabikwa (2020), only one main local equity indicator exists for local clinical commissioning groups (potentially avoidable emergency admissions). This systematic lack of clarity and direction highlights a lack of, and need for, a clear definition for what constitutes a health inequality. NHS Scotland, the King’s Fund and NHS England define health inequalities as unjust and avoidable differences in people’s health across the population and between specific population groups (NHS, n.d.a; Public Health Scotland, 2022; Williams et al., 2020). However, within the existing literature, health inequalities are defined variably by Whitehead (1992) as ‘Social inequities in health are systematic differences in health status between different socio‐economic groups’, by Graham (2009) ‘systematic differences between more and less advantaged groups’, and by Krieger (2001) ‘health disparities, within and between countries, that are judged to be unfair, unjust, avoidable and unnecessary’. The need for a clear definition of health inequality is important for local healthcare partnerships to be able to effectively address them, particularly as the effects of the COVID‐19 pandemic continue to highlight and exacerbate existing health inequalities (Public Health England, 2020).

Hitherto, there exists little evidence that local healthcare partnerships, specifically clinical commissioning groups, have achieved any noteworthy impact on the reduction of health inequalities (Regmi & Mudyarabikwa, 2020). A systematic review of peer‐reviewed papers assessing the barriers and enablers of health inequalities asserts that community‐based targeted and integrated approaches would both improve population health and reduce health inequalities (Regmi & Mudyarabikwa, 2020).

Whilst a lack of a clear definition for health inequality makes it difficult for local healthcare partnerships to address, there are various existing international frameworks and tools they can use to do so. For example, the Place‐Based Approaches for Reducing Health Inequalities framework by Public Health England (2019) emphasises the use of civic‐, service‐ and community‐centred approaches can be used collectively to reduce health inequalities and advance research, respectively. Additionally, Kilbourne et al. (2006) assert that public health interventions should be framed to engage communities, providers and policy makers in order to better understand and tackle determinants of health disparities. More recently, NHS Providers have developed a toolkit for NHS organisations to address inequalities (NHS Providers, 2020).

Currently, there is little research exploring how local healthcare systems conceptualise and approach health inequalities. Here, we analyse local healthcare planning policy documents to explore how local healthcare systems conceptualise health inequalities and possible underlying reasons.

## 2 MATERIALS AND METHODS

We used a document analysis approach to explore how local healthcare systems conceptualised health inequalities within their LTP response documents. Three researchers searched for LTP response documents which are publicly accessible by searching the NHS website and the websites of the local healthcare partnerships such as sustainability and transformation partnerships (STPs) and integrated care systems (ICSs) ([Link], [Link]HS England, n.d.b, n.d.c). Furthermore, a comprehensive Google search was also conducted using the name of the STP with the phrases ‘long term plan,’ ‘long term plan response,’ and ‘5‐year plan’. All of the plans found in the public domain were saved and uploaded into NVivo, version 12.6.

The thematic analysis framework provided by Braun and Clarke (2006) was used to structure the methodological approach of this study. This procedure was applied with the goal of reaching thematic saturation. Five documents were initially read to get familiar with their structure and contents and analysed to test and refine the analysis approach. Sections dedicated to addressing health inequalities were coded. To ensure comprehensive analysis, documents were also searched using key terms (See Appendix 1). This list was drawn from the policy experience within the research team and refined throughout the analysis. After the five test documents were analysed, they were re‐visited a second time for a more thorough analysis. The remaining eight documents were then analysed by the principle coder, and relevant sections of text were coded as appropriate. Three documents were independently double coded by a senior researcher to ensure quality and consistency. New codes were developed continuously throughout the analysis until thematic saturation was reached.

After coding was complete, the codes were analysed for patterns and grouped into candidate themes. Thematic maps were generated to explore the relationship between specific codes and candidate themes.

The candidate themes were reviewed to ensure there were enough data to support them and that each theme was distinct from one another. Candidate themes were synthesised together with the generation of subthemes where necessary. To ensure the distinction between themes, Patton’s (1990) theory of internal homogeneity and external heterogeneity was applied—all codes within a theme were related, and each theme stood independently of the others. Next, defined themes and thematic maps were finalised, and an understanding of how they fit together to answer the research question was established.

## 3 FINDINGS

Thirteen documents were found from a possible 44. The plans were published in September 2019 (*n* = 4), October 2019 (*n* = 1), November 2019 (*n* = 4), December 2019 (*n* = 1) and January 2020 (*n* = 1), with the remaining plans (*n* = 2) not specifying their publication date. The average length of the plans was 167 pages (range: 41–273 pages) and there was good geographical coverage across a broad range of England’s local health organisations such as STPs and ICSs. Of the 13 plans analysed, four contained a chapter addressing health inequalities and prevention and one contained a chapter dedicated solely to health inequalities. The remaining documents combined plans to address health inequalities with more generic plans for prevention or had their health inequalities plan scattered throughout in multiple places within the documents.

**FIGURE 1:**
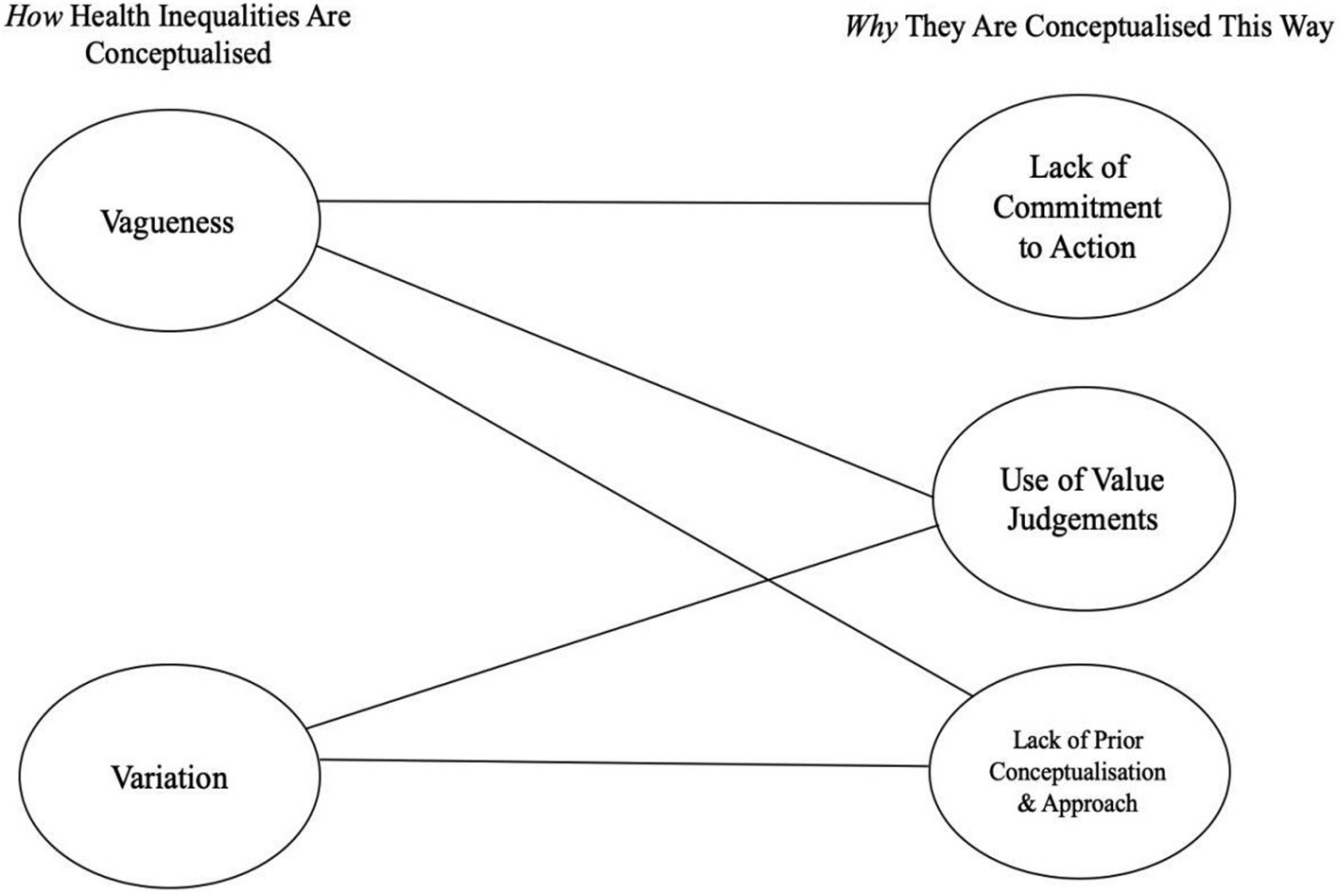
Map of themes

Five themes were identified from the documents; *vagueness* and *variation* described how health inequalities were conceptualised in a way that lacked depth, detail and clarity and *lack of commitment to action*, *use of value judgements* and *lack of prior conceptualisation and approach* explain why they were conceptualised in this way (Figure 1).

### 3.1 How do local health systems conceptualise health inequalities?

#### 3.1.1 Vagueness

Documents were vague and unstructured in their approach to address health inequalities, lacking logical frameworks or schematics. Vagueness was also exemplified by a lack of detail of the key healthcare and health outcome inequalities within and across different groups. This lack of detail and direction led to a lack of clear goals to reduce health inequalities.

The documents contained ambitions for the future albeit vague, undefined and non‐specific, lacking metrics, time frames or a clear description of the populations being discussed or inequalities being targeted.

Vagueness in the documents was also typified by a lack of detail. This was namely found through lack of detailing what health inequalities exist (which health outcomes and in which population groups). Terms found within the plans that came up most often in reference to health inequalities but were used vaguely included ‘health inequality’, ‘outcome’, ‘vulnerable’, ‘hard to reach’ and ‘disadvantaged’. For example, the quote below highlighted the need for improvements in health outcomes to reduce inequalities but did not detail which outcomes these are:

‘By March 2024, the target populations identified in the health and well‐being strategy will be prioritised for action across the System… Improvements in health outcomes should be occurring greater pace in this group than the general population to enable reductions in health inequalities’.(Plan H)

These instances provided evidence that the local response plans acknowledge the LTP’s request to address health inequalities but reflected a superficial conceptualisation by local healthcare systems.

In many instances, when more detail was given in this regard, it was through exemplary language rather than stating the precise outcomes or populations associated with a particular inequality; brief examples were given preceded by the phrases ‘for example’, ‘such as’ and ‘like’. The quote below shows this use of examples:

‘Variation in practice will be removed and efforts directed towards tackling inequalities by focusing on vulnerable groups and those who experience the poorest health outcomes. For example, increasing breastfeeding rates is one of the simplest ways of reducing health inequalities and benefits both women and babies’.(Plan J)

In this way, the local healthcare systems did not paint a full image of the inequalities their respective areas face and how they will be addressed.

#### 3.1.2 Variation

Considerable variation throughout the documents was demonstrated in three ways: variation in definitions of terms, groups being compared and the use of metrics and indicators.

Across the documents, many terms were consistently used in the local health plans’ discussions of health inequalities, however, in different ways. The most common examples of this were found in inconsistent use of ‘at‐risk’ and ‘high‐risk’ groups and how access was defined.

‘At‐risk’ or ‘high‐risk’ groups were inconsistently defined across the documents to include LGBTQ+, BAME (Black, Asian and Minority Ethnic groups), people with mental health problem, black men, rough sleepers and individuals with a disability. Examples of this included:

‘Around one in five adults in [Plan A] smoke, but this increase to at least one in four in the most deprived neighbourhoods as well as in high‐risk groups (e.g. people with mental health problem)’. (Plan A)

‘The council and partners recognise the central importance of evidence‐based parenting support in ensuring school readiness, especially within deprived populations and high‐risk groups and a set of outcome‐based KPIs have been agreed to ensure progress in this area’.(Plan B)

Variation in how health inequalities are conceptualised was also found in how groups are compared to one another. This variation was found both across and within documents. Local health outcomes and risk factors were compared between local populations, within local populations and/or to regional or national averages. For example:

‘Life expectancy varies between our six local places and also within our neighbourhoods’.(Plan E)

‘There is a higher number of premature deaths of people with serious mental health problem compared to the national average’.(Plan K)

Lastly, there was variation in how local healthcare systems used indicators and metrics (outcomes, population groups and time frames used). Most plans made minimal or no use of metrics and indicators to set clear goals for themselves, whilst others were more specific. For example:

‘Meet continuity of carer targets of 35% by March 2020, 51% in 2021 and 75% in 2024 for BAME women and those most vulnerable’.(Plan A)

‘Reduce current variation and inequalities in delivery and health outcomes’.(Plan M)

### 3.2 Why have local health systems conceptualised health inequalities this way?

#### 3.2.1 Lack of prior conceptualisation and approach

Three themes were outlined to answer why health inequalities were conceptualised in the local plans with such vagueness and variation. The first theme is ‘lack of prior conceptualisation and approach’. Documents suggested that local healthcare systems did not have an established approach or work programme prior to the directives published in the LTP. The documents, for instance, frequently mentioned gaps in awareness of what inequalities are present. For example, a lack of awareness of inequalities experienced by minority ethnic groups was shown in the quote below:

‘Improve recording of ethnicity and other protected characteristics in NHS patient records’ in an effort to gather more data surrounding health inequalities experienced by BAME populations in order to reduce them.(Plan K)

The above example demonstrated how lacking an awareness of where health inequalities hindered local healthcare systems’ ability to create detailed plans to reduce health inequalities.

#### 3.2.2 Use of value judgements

The second theme outlined to answer why health inequalities were conceptualised vaguely and variably is ‘use of value judgements’. This was found in the widespread discussion of lifestyle and behaviour being a major determinant of health throughout the documents, as well as in how certain populations were more frequently included, whilst others were consistently left out. The example below highlights this focus on self‐management of lifestyle and behavioural practices as a means of reducing health inequalities, without mentioning a need to address the systematic political, social and environmental origins of health inequalities.

‘The Medicines Optimisation and Pharmacy Transformation (MOPT) programme will work with all health and social care professionals to ensure…Easy access to health and medicines advice to empower residents and patients to control their own health or disease, reducing health inequalities’.(Plan G)

In addition to value judgements being made on the origins of health inequalities, only certain populations were typically addressed. Populations generally discussed were children, people of old age, mothers, the mentally ill, physically disabled and those with autism or learning disabilities. Populations that were consistently left out of the plans included immigrants, minority ethnic groups, criminals and LGBTQ+groups. Additionally, unpaid carers were typically included within the context of how they can help to reduce health inequalities through their work, but there was little discussion of the health inequalities they themselves experience.

#### 3.2.3 Lack of commitment to action

Overall, there was a high level of commitment to the notion of tackling health inequalities, but lack of commitment to take concrete action. This was demonstrated through a lack of concrete and accountable targets or actions. For example:

‘By 2023/24, we will put in place initiatives to tackle health inequalities, with a particular focus on social value, the environment and volunteering’.(Plan J)

Similarly, lack of commitment to action was also found through the widespread and vague use of the term ‘health inequality’ to acknowledge the issue but without a clear plan to do so. For example:

‘Invest levels of resources in developing and implementing a [Plan C] Healthy Lives programme to improve health, reduce health inequalities and reduce the growth in demand for health and care services’.(Plan C)

## 4 DISCUSSION

### 4.1 Overview of findings

Whilst the term ‘health inequalities’ was used throughout the local healthcare system plans, the approach was characterised by variation and vagueness. Health inequalities were conceptualised in the policy response documents in a way that lacked depth, detail and clarity. Use of value judgements, a lack of prior conceptualisation and approach and a lack of commitment in the plans to take action to reduce health inequalities all contributed to the overall vagueness and variation of conceptualisation within and across the plans.

### 4.2 Strengths and limitations

Only 13 of the 44 LTP response plans were identified in the public domain, however thematic saturation was reached. Some of the plans published in the autumn of 2019 may have been revised following feedback by the national inequalities team, so the documents included here may not reflect the final approved documents. However, even early versions of the documents give important data on how local healthcare systems conceptualise health inequalities.

There was good geographic spread within the sample as the local healthcare systems represented by the documents were not geographically clustered in any particular area of the country. Lastly, quality checking and piloting of the analysis procedure ensured a robust analysis.

### 4.3 Implications of the findings

There are a number of possible explanations for the findings. First, the plans appeared to be produced in a rush. Local healthcare partnerships were given a few months to develop a plan to address health inequalities in their respective areas. There were also instances where a plan stated a second document would be released that would explain in greater detail how a particular inequality would be addressed once awareness of the topic was developed further. This could be another indicator of a time constraint to develop the plans. Nevertheless, local healthcare commissioning organisations (Clinical Commissioning Groups) have had a statutory responsibility for the past 6 years to address health inequalities. This, therefore, suggested a lack of prior consideration of health inequalities.

Since the response plans were written to be reviewed by a central healthcare organisation and the language of the plans reflected that of national policy, it is possible that the local healthcare organisations were more focused on using language that would be acceptable to those evaluating the plans, rather than setting out a bold and ambitious plan. This may reflect the dynamic between regional structures and local health systems, whereby local health systems try to mitigate the disruptive impact of national and regional policies on their local priorities.

Another explanation for the vagueness of the local plans is the vagueness of the national LTP document. The national plan itself was lacking in detail, making it less surprising that the subsequent local plans are vague and varied. For example, the National Implementation Framework for the LTP states that an ‘inequalities reduction trajectory’ will be used to monitor the reduction of health inequalities (NHS, 2019). It is not clearly explained if this trajectory will monitor outcomes, risk factors of inequalities, inequalities in access to services or in which groups (Ford et al., 2019). Additionally, there is an overall lack of awareness within the documents of public health frameworks such as the Marmot Review by Marmot et al. (2010) and Graham and Kelly (2004).

Vagueness could have also been due to the value judgement inherent in the definition of health inequalities. These value judgements may be influenced by a strong presence of patient advocates and local champions and the conceptualisation of health inequalities as both the health of marginalised groups (e.g. LGBTQ+) and health changing according to a socio‐economic gradient. This raises the question of what a ‘fair’ inequality is and why some groups are consequently thought of as more ‘deserving’ of ill‐health than others. This mentality only works to perpetuate discriminatory stigma surrounding certain groups which is in of itself unfair.

Whilst a lot of plans did mention social determinants as a cause of health inequality, the plans focused more on self‐management of health, behavioural and lifestyle changes and quality of care; this placed the burden on the individual and focused on a reactive rather than preventative approach. A possible explanation for this focus is that local health systems feel powerless to address the social determinants of health on their own since it requires a collaboration between various cross‐governmental agencies. Emphasising a reactive and lifestyle‐behavioural approach should be cautioned against since it may perpetuate the idea that certain groups are unhealthy because of personal choice.

Lastly, variation across the documents may be useful because it allows for local healthcare systems to determine their own priorities. However, there is a risk that groups with stronger advocate presence are given more attention or higher value judgements, rather than allocating attention based on data‐driven levels of need. For example, the documents prioritise learning disabilities and severe mental illnesses compared to immigrants, refugees, asylum seekers, those with prior justice system involvement and LBGTQ+populations to which little attention was given.

### 4.4 How the findings fit the literature

In terms of the national plan to reduce health inequalities, the findings of this study were in line with criticisms that the plan is vague in scope and lacking in direction to reduce health inequalities (Ford et al., 2019). The findings were also complementary to those of Regmi and Mudyarabikwa (2020) that a more solidified and collaborative approach that is tailored to the needs of each local healthcare partnership is needed to effectively reduce health inequalities (Regmi & Mudyarabikwa, 2020). Some of the findings were consistent with the framework laid out by Graham and Kelly (2004), which outlines that health inequalities could be conceptualised in a variety of ways including poor health of poor people, differences between two groups and as a gradient across populations (Graham & Kelly, 2004). These concepts were mirrored in the variation across and between the plans in the way they talked about health inequalities experienced by deprived or disadvantaged groups, between two groups or both.

It has been argued that the case for groups experiencing inequalities have to be ‘sold’ to non‐academics and policy makers in order for action to be taken, an idea that is in line with our theme of use value judgements (Garthwaite et al., 2016). This need to sell the case to policy makers requires patient representative groups to advocate their case which may be easier for some groups, such as people with learning disabilities or autism. Meanwhile, other groups for which there are less health data on and that are surrounded by more political contention, like immigrants, were consistently left out of the local response plans. This is reflective of findings that migrant health is highly politicised, so health agencies are hesitant to prioritise and allocate resources to this group (Staniforth & Such, 2019). Additionally, there is a little discussion within health inequality policy documents of the relationship between inclusion health groups and the social determinants that lead to their poor health (Tweed et al., 2021). Moving forward, policies to reduce health inequalities would achieve more progress by containing comprehensive conceptual frameworks that take into account social stratification, advantage and disadvantage Tweed et al., 2021).

### 4.5 Policy recommendations

Based on this analysis, we have published a complementary paper which unpacks the concept of health inequalities for local health systems. It sets out to frame inequalities to ensure a systematic and logical approach in health systems, build on long‐term organisational change and redistribute resources and power to prevent illness and promote health (Ford et al., 2021). It sets out the important difference between healthcare inequalities and health outcome inequalities and the differing levels of action: national, system, organisational and individual. We argue that healthcare systems should agree on a systematic and coherent national conceptualisation or framework for health inequalities, such as the one we propose. Any framework should allow local healthcare systems to prioritise according to their local needs, be explicit in differentiating between inequalities in healthcare and health outcomes and contain specified population groups and outcomes measures to focus on. The framework should also ensure that groups which have small local populations, but larger national populations (e.g. the transgender population) are not overlooked. Additionally, it needs to draw on established theorisations of the multi‐faceted nature of health inequalities, such as those used in public health, and be clear in their approach to address both gaps in health outcomes and the health gradient (Sowden et al., 2020).

National conceptualisation needs to go hand in hand with clear guiding principles about how to reduce inequalities. These principles should include the dimensions of inequalities local healthcare systems should consider, and the measures such systems can use to monitor the reduction of inequalities within their jurisdictions. An upskilling of healthcare system decision makers is also needed to promote a more developed approach to inequalities.

A framework also needs to be part of a partnership between multiple governmental bodies to form a wider cross‐government health inequalities strategy to tackle the social determinants of health inequalities (Marmot et al., 2010; Sowden et al., 2020). This partnership approach will be, especially important given the negative impact that COVID‐19 has had on health inequalities and the focus placed on health inequalities in the COVID‐19 recovery plans (Stevens & Pritchard, 2020). Healthcare systems also have an important role in articulating the important link between social factors and health, advocating for geo‐political solutions to address health inequalities and ensure that they use their powers as large employers to improve the social determinants of health. Lastly, the health inequalities’ discourse needs to be expanded on to more often include minority ethnic groups and other population groups that are marginalised, such as immigrants and the LGBTQ+population.

In summary, local healthcare systems conceptualise health inequalities in a vague and variable manner. This may be the result of a lack of prior conceptualisation and approach to understanding of health inequalities, use of value judgements on what fair and unfair health inequalities are and a lack of commitment to take action to reduce health inequalities. These results suggest that the development of an agreed national conceptual framework to guide policy makers and local healthcare decision makers is required to understand and reduce health inequalities.

## CONFLICT OF INTEREST

The authors have no conflicts of interest or relationship, financial or otherwise, to declare that might be perceived as influencing the objectivity or our work.

## AUTHOR CONTRIBUTION

The authors confirm contribution to the paper as follows: study conception and design: J. Ford, J. Olivera; data collection: J. Olivera; analysis and interpretation of results: J. Olivera, J. Ford, S. Sowden, C. Bambra; draft manuscript preparation: J. Olivera, J. Ford, S. Sowden, C. Bambra. All authors reviewed the results and approved the final version of the manuscript.

## Supporting information

Supplementary Material

## Data Availability

The data that support the findings of this study are available from the corresponding author, JNO, upon reasonable request.
