# Supplementary material for: Conceptualisation of health inequalities by local healthcare systems: A document analysis
Source: Health Soc Care Community. 2022 Mar 30;30(6):e3977–84. doi: 10.1111/hsc.13791 (PMC10084138; doi:10.1111/hsc.13791)
Supplement: Supplementary file 1 — Supplementary Material [file HSC-30-e3977-s001.docx]

# Appendix 1: List of Key Search Terms

| **Term of Interest** | **Search Term Used** |
| --- | --- |
| Inequality  Health inequality | Inequa |
| Deprivation  Deprived | Depriv |
| Poverty | Poverty |
| Socio economic | Socio economic |
| Socio-economic | Socio-economic |
| Socioeconomic | Socioeconomic |
| Index of multiple deprivation | Index of multiple deprivation  IMD |
| Adverse childhood experience | Adverse childhood experience  Adverse |
| Ethnic | Ethnic |
| Black and minority ethnic groups | BME |
| Black, Asian and minority ethnic groups | BAME |
| Gay | Gay  Men who have sex with men  MSM |
| Transgender | Transgender |
| LGBTQI+ | LGBT |
| Criminal offenders | Criminal  Prison  Offender  Incarcera |
| Carers | Carers |
| Rough sleepers | Rough sleep |
| Gypsy, roma, travelers | Gypsy  Roma  Traveler |
| Asylum seekers | Asylum |
| Refugees | refugee |
| Immigrants and migrants | Migrant |
| Sex workers | Sex work |
| Severe mental illness | Severe mental illness |
| Learning difficulties  Learning disabilities | Learning di |
| Health Inequities | Inequ |
| Vulnerable groups | Vulnerable |
